# Development of a qPCR Method to Measure Mitochondrial and Genomic DNA Damage with Application to Chemotherapy-Induced DNA Damage and Cryopreserved Cells

**DOI:** 10.3390/biology5040039

**Published:** 2016-10-08

**Authors:** Stephen O. Evans, Michael B. Jameson, Ray T. M. Cursons, Linda M. Peters, Steve Bird, Gregory M. Jacobson

**Affiliations:** 1 Biomedical Research Unit, School of Science, University of Waikato Private Bag 3105, Hamilton 3240, New Zealand; soe2@students.waikato.ac.nz (S.O.E.); rtmc@waikato.ac.nz (R.T.M.C.); lpeters@waikato.ac.nz (L.M.P.); gmjacobson@gmail.com (G.M.J.); 2 Department of Oncology, Regional Cancer Centre, Waikato Hospital, Hamilton West 3204, New Zealand; Michael.Jameson@waikatodhb.health.nz

**Keywords:** LORD-Q, qPCR, comet assay, DNA damage, PBMC, THP1, A549, bleomycin, cisplatin

## Abstract

DNA damage quantitation assays such as the comet assay have focused on the measurement of total nuclear damage per cell. The adoption of PCR-based techniques to quantify DNA damage has enabled sequence- and organelle-specific assessment of DNA lesions. Here we report on an adaptation of a qPCR technique to assess DNA damage in nuclear and mitochondrial targets relative to control. Novel aspects of this assay include application of the assay to the Rotor-Gene platform with optimized DNA polymerase/fluorophore/primer set combination in a touchdown PCR protocol. Assay validation was performed using ultraviolet C radiation in A549 and THP1 cancer cell lines. A comparison was made to the comet assay applied to peripheral blood mononuclear cells, and an estimation of the effects of cryopreservation on ultraviolet C-induced DNA damage was carried out. Finally, dose responses for DNA damage were measured in peripheral blood mononuclear cells following exposure to the cytotoxic agents bleomycin and cisplatin. We show reproducible experimental outputs across the tested conditions and concordance with published findings with respect to mitochondrial and nuclear genotoxic susceptibilities. The application of this DNA damage assay to a wide range of clinical and laboratory-derived samples is both feasible and resource-efficient.

## 1. Introduction

The integrity of genomic and mitochondrial DNA is under constant threat from endogenous reactive oxygen species produced by normal cellular metabolism and exogenous sources such as ultraviolet light and mutagens. To counter this, organisms use a complex network of pathways/processes that sense and repair defects, collectively referred to as the DNA damage response (DDR) [1]. However, loss of function mutations or epigenetic silencing of DDR genes—which are associated with human malignancies and inherited disorders such as Fanconi anaemia and Bloom’s syndrome—can prevent repair of DNA [2]. A reliable method that detects and quantifies DNA lesions is valuable in understanding disease-specific pathologies and screening chemical and environmental contaminants for genotoxic effects.

Quantification of mutagenic effects originated with the micronucleus assay [3], which was largely superseded by the single-cell gel electrophoresis or comet assay [4]. This method quantifies the migration of damaged DNA fragments from the nucleoid under electrophoresis and has become the gold standard for measuring cellular DNA damage. However, researchers are exploring PCR-based approaches, where DNA damage results in the disruption of DNA polymerase activity and impaired DNA synthesis. Early investigations used semi-quantitative PCR approaches with target amplicons of between ~450 bp and 25 kb [5,6], which has been updated to incorporate quantitative PCR (qPCR) [7]; however, due to the multistep validation required, its use is not straightforward. Contemporaneously, the long-run qPCR technique for DNA-damage quantification (LORD-Q) method was developed [8], in which a high frequency of lesions in the DNA template corresponds to a lower concentration of full-length template and therefore a higher threshold value of the amplified product relative to the undamaged template. This technique utilized oligonucleotide primers designed for 3–4 kb target fragments and small internal amplicons of ~100 bp, and was able to quantify DNA damage induced by a range of genotoxic stimuli and interrogate specific DNA loci in both mitochondrial and genomic DNA with reproducible sensitivity. However, this method has not been widely tested on different instruments (the published report used Roche LightCycler 480 II instrument) and has the disadvantage of requiring an expensive DNA polymerase and fluorophore.

Here we report a modified long-run qPCR assay for use on a Corbett Rotor-Gene 6000 instrument, with alternative primer sets for both nuclear and mitochondrial DNA loci. The method allows both small and large products to be amplified under the same PCR conditions using considerably cheaper consumables than the published method. Our approach was validated by measuring DNA damage in: (1) cryopreserved and freshly-isolated peripheral blood mononuclear cells (PMBCs) exposed to ultraviolet C (UVC) radiation (254 nm); (2) suspension and adherent cancer cell lines exposed to UVC; and (3) cryopreserved PBMCs damaged by cytotoxic chemotherapy (cisplatin and bleomycin). 

## 2. Materials and Methods

### 2.1. Cell Culture

THP1 and A549 human cell lines were obtained from ATCC and cultured in RPMI-1640 + GlutaMAX (Gibco, Grand Island, NY, USA), supplemented with 10% heat-inactivated fetal calf serum (FCS, Gibco, USA), penicillin, and streptomycin (Gibco, USA). All cell culture experiments were carried out in a humidified incubator at 5% CO_2_ and 37 °C. Adherent cells were harvested by trypsin incubation using 0.05% trypsin in EDTA (Gibco, USA). PBMCs were extracted from buffy coats collected from healthy blood donors by the NZ Blood Service under ethical approval from the Northern B Health and Disability Ethics Committee (reference NTY/10/08/065/AM01). Buffy coats were diluted with phosphate-buffered saline (PBS, pH 7.4) and layered over Histopaque 1077 (Sigma-Aldrich, St Louis, MO, USA) in 15 mL conical sterile polypropylene centrifuge tubes (Greiner Bio-One, Frickenhausen, Germany, The cells were centrifuged at 400 *g* for 30 min, the interphase removed and washed three times in 10 mL of sterile PBS and centrifuged at 250 *g* to remove residual platelets. Finally, the cells were quantified in a Neubauer chamber (Fortuna, Wertheim, Germany) and viability was assessed by 0.4% *w*/*v* trypan blue exclusion (Sigma-Aldrich) in PBS. Samples were used in the experiments if viability exceeded 85%.

### 2.2. Cryopreservation of PBMCs

PBMCs were resuspended in sterile filtered 50% FCS (Gibco, USA), 40% RPMI (Gibco, USA) and 10% DMSO (Merck, Darmstadt, Germany) in 1.8 mL Nunc™ conical sterile polypropylene cryo vials (Thermo Scientific, Waltham, MA, USA) and placed overnight in a CoolCell^®^ (Biocision, Larkspur, CA, USA) at −80 °C for controlled cooling. These cells were then stored for 2, 4, 8, or 12 weeks at −80 °C. When required, PBMC aliquots were thawed rapidly in a 37 °C water bath, gradually equilibrated with pre-warmed culture media, diluted to 10× the original culture volume, and the cells were pelleted by centrifugation in a refrigerated centrifuge (Eppendorf, Hamburg, Germany) at 300 *g* for 10 min. The residual storage media was discarded to ensure no carryover of DMSO, and the cells were resuspended and maintained in RPMI-1640 + GlutaMAX (Gibco, USA) and supplemented with 10% heat-inactivated FCS (Gibco, USA) in standard culture conditions for 24 h before UVC irradiation or treatment with cytotoxic compounds.

### 2.3. UVC Irradiation of Cells

UVC irradiation was carried out on THP1, A549, and fresh or cryopreserved PBMC cells. Cell concentrations were 2.5–5 × 10^5^/mL for A549 cells, 5 × 10^5^/mL for THP1, and 1 × 10^6^/mL for PBMCs, aliquoted into identical 2 mL media volumes in six-well Nunclon™ Delta plates (Thermo Scientific, USA). UVC doses of either 20 or 100 millijoules per cm^2^ (mJ/cm^2^) were given to each plate using the Bio-link BXL-254 cross-linker (Gibco, USA). Cells were harvested immediately after UVC exposure, and were prepared for DNA extraction, except for aliquots of fresh PBMCs that were used within the alkaline comet assay.

### 2.4. Cytotoxic Chemotherapy Incubation

Bleomycin and cisplatin concentrations were chosen to match those used in a previous study [8]. A 10 mg vial of bleomycin (Hospira, Lake Forest, IL, USA ) was reconstituted in sterile 0.9% *w*/*v* sodium chloride and stocks frozen at −20 °C. Revived PBMCs were resuspended in serum-free media RPMI-1640 (Gibco, USA) in six-well Nunclon™ Delta plates (Thermo Scientific, USA) containing 2.5 × 10^6^ cells per well with bleomycin at concentrations of 10, 20, 30, or 40 μM or vehicle (0.9% *w*/*v* sodium chloride). Plates were incubated for 30 min at 37 °C in 5% CO_2_, after which cells were washed in PBS and prepared for DNA extraction. A 1 mg/mL stock solution of cisplatin (Novartis, Auckland, New Zealand) was diluted to the required concentration in complete media RPMI-1640 (Gibco, USA). Revived PBMCs were resuspended in complete media RPMI-1640 (Gibco, USA) in six-well Nunclon™ Delta plates (Thermo Scientific, USA) containing 2.5 × 10^6^ cells per well with cisplatin at concentrations of 50 or 100 μM or media control. Plates were incubated for 24 h at 37 °C in 5% CO_2_, after which cells were washed in PBS and prepared for DNA extraction.

### 2.5. DNA Extraction

Total cell DNA was isolated and purified using Quick-gDNA^TM^ Miniprep (Zymo Research, Irvine, CA, USA) as per the manufacturer’s instructions from control and treated cells. The cells were washed in PBS and then lysed immediately after UV or chemotherapy treatment by application of 400 μL of the Quick-gDNA^TM^ Miniprep lysis reagent to adherent cells directly on the plate or to suspension cells after 5 min centrifugation at 200 *g*. DNA eluted in TE buffer (10 mM Tris-HCl/1 mM EDTA) was quantified, and an assessment of DNA purity was determined on a NanoDrop 2000 (Thermo Scientific, USA) by spectrometric analysis. An aliquot of the high quality DNA (A260:280 > 1.8) was subsequently stored at 4 °C.

### 2.6. Alkaline Comet Assay

Fronine microscope slides (Thermo Fisher, Taren Point, Australia) were cleaned, coated with molten 1% HyAgarose™ LE Agarose (Hydragene, Xiamen, China) by emersion, and air-dried. Experimentally-treated cells at a concentration of 1 × 10^4^ cells per slide were mixed into 37 °C 0.5% low melting point (LMP) SeaPlaque™ GTG™ Agarose (FMC Bioproducts, Rockland, ME, USA) in PBS and pipetted onto the agarose-coated slides. A 15 × 40 mm coverslip was immediately placed on the LMP agarose/cell solution, and the slides were left to gel at 4 °C in the dark for 20 min. Next, the slides were placed in comet lysis solution (pH 10; 2.5 M sodium chloride, 100 mM EDTA, 1.2 mM Tris-HCl, and 0.1% SDS), with 0.015% Triton X-100 added immediately prior to use, for 2 h at 4 °C. Slides were then washed in cold PBS for 5 min and then placed in an Owl™ D3 Electrophoresis System chamber (Thermo Scientific, USA) containing approximately 650 mL of alkaline electrophoresis buffer (pH > 13; 300 mmol sodium hydroxide, 1 mmol disodium EDTA) for 30 min in the dark. Then, electrophoresis was carried out at 300 mA and 20 V for 20 min, after which slides were carefully removed from the chamber and washed three times in neutralization buffer (Tris 0.4 M; pH 7.5) at 4 °C. Cells were stained with SYBR gold fluorophore 10,000× concentrate (Life technologies, USA) diluted in distilled water for 10 min, and excess stain was washed off with two 5 min distilled water washes. Comet images were captured at 100× magnification using a Leica I3 filter block and Olympus DP70 camera. Slides were prepared in triplicate, and 50 cells were scored per slide for each experimental condition. Olive tail moment was calculated for each cell using CometScore analysis software (Tritek Corp, Sumerduck, VA, USA).

### 2.7. Modified Long-Run qPCR Technique for DNA-Damage Quantification

Four oligonucleotide primer sets were designed using Primer3 [9] for a long nuclear gene target of 3129 bp in the E2F transcription factor 1 (E2F1) gene (accession no: AF516106.1; 3427–6565 bp) and a long mitochondrial target 3723 bp (accession no: NC_012920; 11492–15214 bp). For each long amplicon target, a matched reverse primer for an internal short amplicon of between 50 and 150 bp was also designed (Table 1). All primer sequences were tested using the primer blast web tool (http://www.ncbi.nlm.nih.gov/tools/primer-blast/) to ensure primer specificity.

qPCR analysis was performed using a Corbett Rotor-Gene 6000 instrument (Corbett Research, Mortlake, Australia). All reaction components were optimized through extensive trialing. Final conditions were: 1× buffer B1, 2.5 mM MgCl_2_, 1 U Hot FIREPol^®^ DNA Polymerase (Solis Biodyne, Tartu, Estonia), 200 µM dNTPs (Genscript, Piscataway, NJ, USA) and 2 µM SYTO82 fluorophore (Life Technologies, USA) in a 20 µL reaction volume, which also contained primer at a concentration of 500 nM and 25–50 ng of DNA template (diluted in MQ sterile water). Each reaction was carried out in an Axygen^®^ optically-clear thin-walled PCR tube (Corning, Tewksbury, MA, USA) in a 36-well rotor. The optimized cycling conditions (Table 2) included a Hotstart step (10 cycles with a 0.5 °C decrease per cycle) and an extension of 240 s to ensure amplification of the long product.

PCR amplification efficiencies for the target amplicons were calculated by comparative quantitation using the Corbett Rotor-Gene 6000 Application Software, version 1.7 (Qiagen,Valencia, CA USA). Average efficiencies for both large and small products across each experimental series were used for these calculations. Detected lesion rate per 10 kb was determined using the following equation modified from Lehle et al. [8]:

Lesions per 10 kb = [(E*_L_*^C*pl*(Sample)^ × E*_S_*^−C*ps*(Sample)^/E*_L_*^C*pl*(Control)^ × E*_S_*^−C*ps*(Control)^)^1/*a*^ − 1] × 10,000

where E*_L_* and E*_S_* are the average amplification efficiencies of the large and short product, *C_p_* values are the crossover (or threshold) values determined by the Rotor-Gene software (*C_pl_*: long; C*_ps_*: short), and *a* is the number of base pairs of the long fragment. All qPCR reactions for each sample were carried out in duplicate, and the mean *C_p_* values were used in the calculation of lesions in either genomic or mitochondrial DNA. An Excel spreadsheet for the above calculation is included in the supplementary materials (Table S1).

### 2.8. Data Analysis

Graphs were constructed using Prism v7 (GraphPad Software, La Jolla, CA., USA), with data expressed as mean ± SE. Results of the qPCR and comet assays were tested using the unpaired *t*-test and two-sided *p* values < 0.05 were considered significant. One-way ANOVA comparison was used to test differences between means at different time points.

## 3. Results

### 3.1. Adherent and Suspension Cancer Cell Lines DNA Damage Quantitation

**Figure 1:**
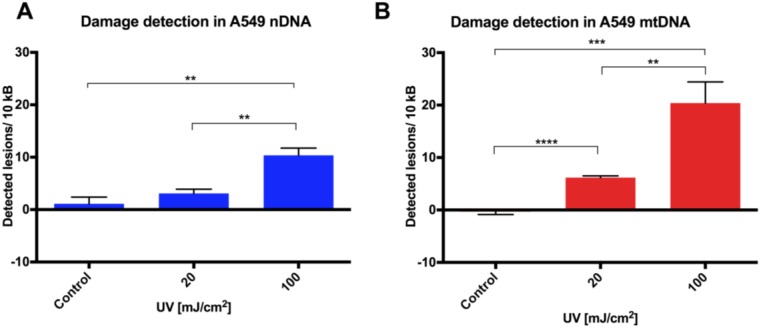

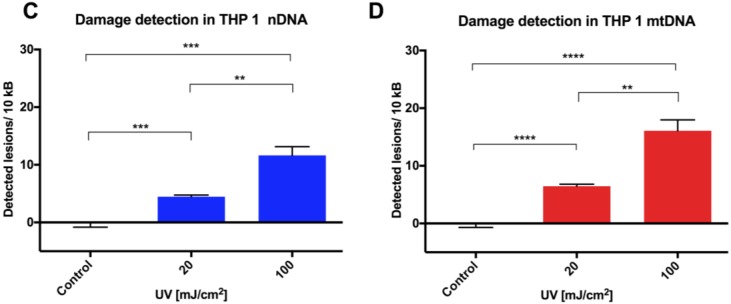
Nuclear DNA (nDNA) and mitochondrial DNA (mtDNA) damage quantitation in cancer cell lines. Genotoxic stimulation with UVC (20 or 100 mJ/cm^2^) was assessed in adherent A549 cells in (**A**) nDNA and (**B**) mtDNA, and in the THP1 suspension cell line in (**C**) nDNA and (**D**) mtDNA using qPCR (*n* = 5). Results are presented as mean ± SE. ** *p* <0.01, *** *p* < 0.001, **** *p* <0.0001.

After 20 or 100 mJ/cm^2^ UVC (254 nm) was applied to the A549 human lung cancer cell line and DNA extracted; qPCR was performed on both long and short fragments for both nuclear DNA (nDNA) and mitochondrial DNA (mtDNA) using our optimized touchdown protocol. Each experiment was performed in triplicate, and results obtained from two independent investigations. DNA lesion rates were higher in the mitochondrial than in nuclear DNA templates, with a clear dose response to UVC exposure in both target amplicons (Figure 1A,B). Due perhaps to variation in C*_p_* values for the E2F1 fragment between controls, there was no statistically significant nDNA response at 20 mJ/cm^2^. A similar pattern of significant DNA damage from both 20 and 100 mJ/cm^2^ UVC was seen in the THP1 human monocytic leukemia cell line (Figure 1C,D).

### 3.2. DNA Damage Quantitation in PBMCs Using qPCR and the Comet Assay

**Figure 2:**
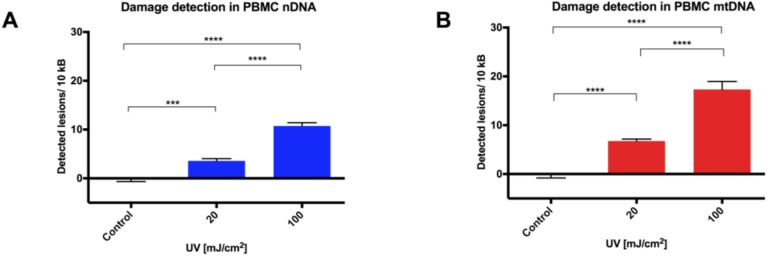

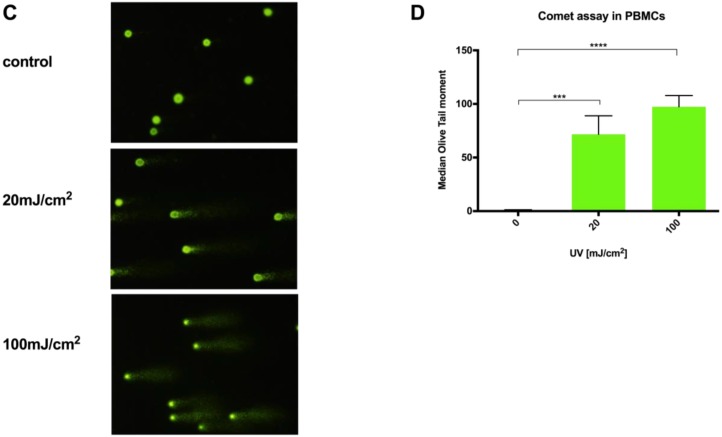
Comparison of the qPCR approach to the alkaline comet assay using peripheral blood mononuclear cells (PBMCs). Genotoxic stimulation with ultraviolet C (UVC; 20 or 100 mJ/cm^2^) was assessed in freshly isolated PBMCs in (**A**) nDNA and (**B**) mtDNA by qPCR (*n* = 9). DNA damage was also assessed after the same treatments using (**C**) the alkaline comet assay, and (**D**) median Olive tail moments were calculated for each slide (*n* = 9). Results are presented as mean ± SE. *** *p* < 0.001, **** *p* < 0.0001.

PBMCs isolated from healthy donor buffy coats were equilibrated overnight in complete media and then treated with UVC. Aliquots were then taken for either the alkaline comet assay or DNA extraction for qPCR. Experiments were performed in triplicate from three different PBMC donors (*n* = 9). qPCR revealed a UVC dose response in both nDNA (Figure 2A) and mtDNA (Figure 2B) across the nine replicates. While the comet assay was able to distinguish between control and UVC-exposed cells (Figure 2C), it failed to discriminate between 20 and 100 mJ/cm^2^ doses, with significant variation in Olive tail moment observed (Figure 2D). The authors who first reported the LORD-Q approach also demonstrated the superiority of qPCR over the alkaline comet assay in detecting low-level DNA damage induced by exposure to bleomycin in Jurkat T cells [8].

### 3.3. Effect of Cryopreservation on DNA Damage Quantitation in PBMC’s

**Figure 3:**
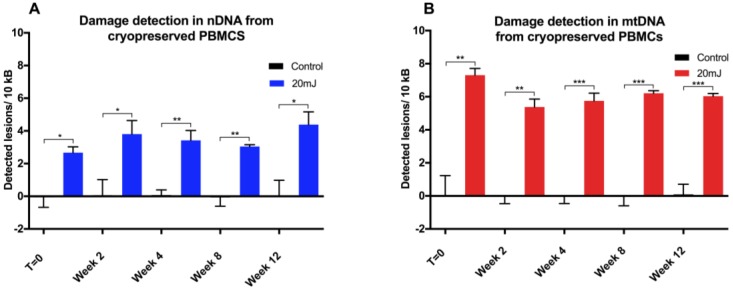
DNA damage assessment in cryopreserved PBMCs. qPCR measurement of UVC-induced (**A**) nDNA and(**B**) mtDNA damage in revived PBMCs following cryopreservation over 12 weeks (*n* = 3). Results are presented as mean ± SE. * *p* < 0.05, ** *p* < 0.01, *** *p* < 0.001.

The use of the qPCR assay in quantifying UVC-induced DNA damage in cryopreserved PBMCs was assessed (Figure 3). Experiments were performed in triplicate from one PBMC donor (*n* = 3). For the nuclear DNA target (*E2F1*), the mean lesion rates observed across five time points from *t* = 0 (fresh) to *t* = 12 weeks at −80 °C after treatment with 20 mJ of UVC were not significantly different (*p* = 0.36). In contrast, induced DNA damage in the mitochondrial fragment was significantly greater at *t* = 0 (mean 7.3 lesions/10 kb) than at subsequent time points (*p* = 0.034). We observed that no further deterioration in DNA integrity was found over a 12 week period in either mitochondrial or nuclear DNA, with cells stored for different periods of time giving similar DNA damage response to UVC challenge.

### 3.4. DNA Damage Quantitation in PBMCs Exposed to Cytotoxic Drugs

**Figure 4:**
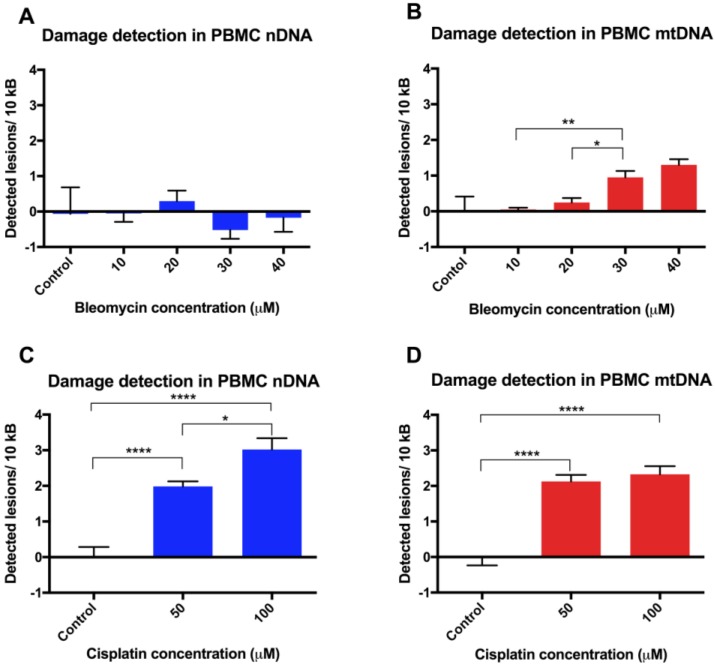
Cytotoxic chemotherapy-induced DNA damage in PBMCs. Quantitation of DNA lesion rates in revived PBMCs in (**A**) nDNA and (**B**) mtDNA following exposure to bleomycin or in (**C**) nDNA and(**D**) mtDNA following exposure to cisplatin (*n* = 6). Results are presented as mean ± SE. * *p* < 0.05, ** *p* < 0.01, ***p < 0.001, **** *p* < 0.0001.

PBMCs revived after cryopreservation (storage period <4 weeks for all PBMC aliquots) were treated with either the cytotoxic glycopeptide antibiotic bleomycin (Figure 4A,B), the platinum compound cisplatin (Figure 4C,D), or media as a control. Experiments were performed in triplicate from two different PBMC donors (*n* = 6). Initial experiments using bleomycin on PBMCs showed no significant nDNA damage, and a slight significant increase in mtDNA damage. Due to these findings, no more replicates were performed, and another drug was tested for comparison. Interestingly, a higher frequency of DNA lesions was observed in nDNA than mtDNA in PBMCs treated with 100 μM cisplatin (Figure 4C,D), both being greater than for bleomycin treatment.

## 4. Discussion

Current techniques available to measure DNA damage (such as the comet assay) can be both time-consuming and laborious when applied to large sample numbers. There are increasing reports in the literature of methods that utilize molecular techniques like qPCR, which have the potential to reduce analysis time and offer a significant level of sensitivity and reproducibility. They also improve on the comet assay with sequence-specific DNA damage, quantifying the effects of potential toxicants separately on nuclear and mitochondrial DNA.

Initially, we evaluated the LORD-Q method using primer sets as previously described [8], testing for amplification of both the large nuclear gene (*p53*) (3075 bp) and large mitochondrial (3723 bp) products. qPCR was carried out using the recommended KAPAG2 (Peqlab) enzyme and cycling conditions with the SYTO82 fluorophore using a Corbett Rotor-Gene 6000 (Qiagen). We also tried substituting the enzyme for the FIREPol^®^ DNA Polymerase (Solis BioDyne), but the amplification gave variable results with a number of non-specific products. This was particularly evident for the p53 product, as confirmed by gel electrophoresis. Through a series of investigations, we eventually optimized this assay by targeting different DNA regions, using a less expensive HotFirePOL enzyme/SYTO82 fluorophore combination (approximately six times cheaper than the LORD-Q approach) and establishing the best cycling conditions for use on our own qPCR system. We chose *E2F1* as a nuclear DNA target, as this gene plays a key role in cell cycle regulation, is likely to be in a transcriptionally-active region of the genome, and is therefore susceptible to experimentally-induced damage [10]. We anticipate that there would be a multitude of suitable targets throughout the genome, and that some optimization would be required when using different qPCR instruments. The reproducibility of this modified qPCR assay was validated using UVC treatment (254 nm) initially in adherent (A549) and suspension (THP1) cancer cells lines, then in PBMCs due to a forthcoming clinical investigation involving such samples. Moreover, the comparison between levels of induced DNA damage in cryopreserved versus freshly-isolated PBMCs informs future experimental methodologies and clinical trial material handling protocols.

Initial assay validation using UVC to damage THP1 and A549 cell lines demonstrated reproducible damage quantification in both nDNA and mtDNA, which was very close to what had initially been shown in Jurkat T-cells in the LORD-Q study [8]. Alternative approaches to measuring DNA damage in THP1 cells have shown similar UVC-induced damage. In addition to inducing hydrolytic and oxidative damage, UVC induces bipyrimidine sites in cellular DNA to form cyclobutane pyrimidine dimers, (6–4) photoproducts and their Dewar isomers [11]. Using high performance liquid chromatography with electrospray ionization tandem mass spectroscopy (HPLC-MS/MS), the UVC-induced bipyrimidine lesion rate in THP1 cells was approximately 1 lesion per 1 × 10^4^ base pairs per 10 mJ/cm^2^ at a cell concentration of 6 × 10^6^/cm^2^ [12]. Interestingly, A549 and THP1 cells had much higher damage in mtDNA than nDNA, which was similar to another study, where Jurkat T cells had been damaged by UVC [8]. Mitochondrial DNA has been shown to be more sensitive to oxidative stress than nDNA [13], perhaps related to its proximity to a major source of endogenous reactive oxygen species, namely the electron transport chain located in the inner mitochondrial membrane. Furthermore, the nucleotide excision repair pathway responsible for repairing helix-distorting lesions (such as UVC-induced photoproducts) is absent in mitochondria [14].

While the comet assay is still regarded as the standard genotoxicity assay, it does have a number of limitations. Firstly, standardization of assay conditions is vital in reducing intra-assay variability [15]; even small changes in agarose concentration, alkaline unwinding times, and electrophoresis conditions can significantly alter the results. Additionally, even with the use of comet scoring software, bias can be introduced into the scoring process and, although the comet can be scaled up to deal with large sample numbers, significant time resources are required to perform the assay, then capture and score the images. In comparison, a qPCR-based technique provides a relative rather than absolute measurement of DNA damage. It allows differences in mitochondrial and nuclear damage to be compared, and can provide gene/region-specific information that may clarify our understanding of certain pathological processes. To our knowledge, this report is the first comparison of a qPCR-based DNA damage assay with the comet assay in PBMCs. The results show our optimized protocol to be a suitable alternative technique to the comet assay in quantifying genotoxicity from UVC exposure in PBMCs in a time and reagent cost-efficient manner.

The use of human lymphocyte-based model systems for screening chemical compounds for cytotoxicity or cytoprotection is well-established. The cryopreservation of lymphocytes enables long-term storage and allows down-stream assays (such as toxicity assays) on cells collected over an extended period of time, such as in a clinical study. The study of DNA damage and repair has been performed in cryopreserved PBMCs using the comet assay [16]. Whilst the isolation of PMBCs from whole blood is associated with some DNA damage, the additional cryopreservation step should not contribute to further damage, with responses following the induction of oxidative stress being similar between frozen and fresh lymphocytes [17]. Indeed, we observed that no further deterioration in DNA integrity was found in either mitochondrial or nuclear DNA over a 12 week period, with cells stored for different periods of time giving similar DNA damage response to UVC challenge. The utility of this approach has been demonstrated here in both fresh and in cryopreserved PBMCs, and can be applied to the analysis of tissue-banked samples in a high throughput qPCR format.

Finally, assay validation using the cytotoxic chemotherapy agents bleomycin and cisplatin in revived PBMCs revealed variable susceptibility to their genotoxic effects. The observed lower levels of damage in nDNA in unstimulated PBMCs is not unexpected, given that previous studies on DNA damage following a 30 min exposure of phytohemagglutinin-stimulated PBMCs to bleomycin 20 µg/mL have shown a very low level of damage, as assessed by the alkaline comet assay, with <1% DNA in the tail [18]. A semi-quantitative PCR approach also showed nDNA from SV40-transformed human fibroblasts to be more sensitive than mtDNA to damage following incubation with cisplatin [19]. Other PCR-based studies have been undertaken using cisplatin, where DNA adducts were measured in a p53 nuclear DNA target in donor PBMC cell lysates [20], and a lesion frequency of ~1.2/104 nucleotides was estimated after a 3 h treatment with 100 μg/mL cisplatin. However, this is not directly comparable to our data, where cisplatin was used at lower concentrations for 24 h incubations and PCR was performed on high quality extracted DNA rather than directly on cell lysates. 

## 5. Conclusions

The successful assay implementation of a qPCR assay to measure nDNA and mtDNA damage requires optimization of primers from selected targets and qPCR cycling conditions for a given PCR platform and experimental system/cell line. Our comparison of induced DNA damage in fresh and cryopreserved PBMCs provides the basis for future applications, such as human bio-monitoring studies of bio-banked samples. This assay is recommended as a useful and versatile tool to assess DNA damage within genomic and mitochondrial DNA in any eukaryotic system, even in the same PCR run across different experimental groups.

## Figures and Tables

**Table 1:** Primers designed to amplify large and small products for E2F1 and the mitochondrial targets.

Primer Name	Temp. (°C)	Sequence	Product Size (bp)
E2F1 large forward	65.2	GAGGCAGGACTCAGGACAAG	3129
E2F1 large reverse	65.2	CTCCTCACATGCAGCTACCA	
E2F1 small reverse	65.3	GGATGCCTCAGGGACCAG	164
Mitochondrial large forward	63.6	CGCCTCACACTCATTCTCAA	3723
Mitochondrial large reverse	62.6	AATGTATGGGATGGCGGATA	
Mitochondrial small reverse	62.9	CAAGGAAGGGGTAGGCTATG	55

**Table 2:** Optimized cycling conditions for quantitative polymerase chain reaction (qPCR).

Cycle Step	Incubation Times
Initial denaturation (1 cycle)	95 °C at 15 min
Hot start (10 cycles)	Step 1: 95 °C at 10 s Step 2: 65 °C (−0.5 °C/cycle) at 10 s Step 3: 72 °C at 240 s
Amplification (35 cycles)	Step 1: 95 °C at 15 s Step 2: 60 °C at 15 s Step 3: 72 °C at 240 s acquiring to cycling A (yellow channel) Step 4: 82 °C at 10 s acquiring to cycling B (yellow channel)
Melt curve	Ramp from 64 °C to 95 °C Hold for 90 s on the 1st step Hold for 5 s on the subsequent steps. Melt A (yellow channel)

## Supplementary Materials

The following are available online at www.mdpi.com/2079-7737/5/4/39/s1, Table S1: qPCR DNA damage calculator.
